# Effects of Hyperthyroidism on Venous Thromboembolism: A Mendelian Randomization Study

**DOI:** 10.1155/2022/2339678

**Published:** 2022-10-12

**Authors:** Fushi Han, Chunyang Zhang, Miao Xuan, Zhuangli Xie, Kunming Zhang, Ying Li

**Affiliations:** ^1^Department of Nuclear Medicine, Tongji Hospital, Tongji University School of Medicine, Shanghai 200065, China; ^2^Department of Endocrinology, Tongji Hospital, Tongji University School of Medicine, Shanghai 200065, China; ^3^Department of Oncology, Tongji Hospital, Tongji University School of Medicine, Shanghai 200065, China

## Abstract

**Objective:**

Observational studies show the correlation between thyroid dysfunction and risk of venous thromboembolism. However, the causal effects remain uncertain. Our study was conducted to evaluate whether thyroid function and dysfunction were causally linked to the risk of venous thromboembolism.

**Methods:**

Publicly available summary data of thyrotropin (TSH) and free thyroxine (FT4), hypothyroidism, and hyperthyroidism were obtained from the ThyroidOmics Consortium and the UK Biobank. With single nucleotide polymorphisms (SNPs) as instrumental variables, the casual effects of genetically predicted TSH and FT4 and hypo- and hyperthyroidism on venous thromboembolism outcome were estimated through Mendelian randomization analysis methods (inverse variance weighted (IVW), MR-Egger, weighted median, simple mode, and weighted mode). Cochran's Q test was performed to evaluate the heterogeneity and horizontal pleiotropy.

**Results:**

Our study selected 15 FT4-, 36 TSH-, 3 hyperthyroidism-, and 79 hypothyroidism-associated SNPs as instrumental variables. The IVW analysis results showed that the odds ratio of venous thromboembolism for hyperthyroidism was 1.124 (95% confidence interval: 1.019-1.240; *p* = 0.019), demonstrating the casual effect of hyperthyroidism not FT4, TSH, and hypothyroidism on venous thromboembolism. No heterogeneity or horizontal pleiotropy was observed according to Cochran's Q test.

**Conclusion:**

Our Mendelian randomization analysis supports the causal effect of hypothyroidism on risk of venous thromboembolism. There is no evidence that genetically predicted TSH, FT4, and hypothyroidism have casual effects on venous thromboembolism. Future studies should be conducted to elucidate the underlying pathophysiological mechanisms.

## 1. Introduction

Venous thromboembolism, including deep vein thrombosis and pulmonary embolism, is a chronic disease that affects approximately 10 million individuals per year globally [1, 2]. The annual incidence of acute venous thromboembolism is 1-2 cases per 1000 people and is four times higher in high-income countries compared with low-income countries [3]. The diagnostic methods of venous thromboembolism consist of sequential examinations combining clinical probabilities, D-dimer test, and imaging assessments [4]. Venous thromboembolism therapy is aimed at preventing thrombus extension and embolism, cardiopulmonary failure, deaths, recurrence, and long-term complications [5]. Direct oral anticoagulants and thrombin inhibitor are the major treatment strategies in the management of venous thromboembolism [6]. Venous thromboembolism is a multifactorial illness induced by the interaction of multiple predisposing factors [4]. Observational studies have showed the correlation between thyroid dysfunction and risk of venous thromboembolism. A prospective cohort study showed that hyperthyroidism not hypothyroidism was associated with lower risk of recurrent venous thromboembolism among elderly patients [7]. A meta-analysis of cohort studies also showed that hyperthyroidism was a risk factor of venous thromboembolism [8]. However, in a retrospective study, increased risk of venous thromboembolism was observed among patients with hypothyroidism not hyperthyroidism [9]. Nevertheless, it remains unclear whether the above observed associations are causal, because observational studies could have selection bias, residual confounding, and reverse causality.

Mendelian randomization is a research method that uses genetic variation as an instrumental variable to establish a model to test the causal relationship between variable exposure factors and diseases [10, 11]. Single nucleotide polymorphism (SNP) loci discovered by genome-wide association study (GWAS) based on large samples provide a large number of genetic variation tool variables for Mendelian randomization analysis, which can effectively reduce the bias of causality estimates [12–14]. Mendelian randomization has been widely used in venous thromboembolism studies, revealing the intrinsic link between various phenotypes and venous thromboembolism [15–17]. For instance, a Mendelian randomization study showed that obesity is casually correlated with venous thromboembolism [18]. Another study found that taller height acts as a risk factor of venous thromboembolism [19]. Based on previous research, this study collected currently available GWAS data on thyroid function and dysfunction. The causal relationships of free thyroxine (FT4), thyrotropin (TSH), hyperthyroidism, and hypothyroidism with venous thromboembolism were assessed using two-sample Mendelian randomization, which might provide a basis for prevention and treatment of venous thromboembolism.

## 2. Materials and Methods

### 2.1. Summary Data and Study Population

Our study acquired publicly available genetic summary data from two large GWAS cohorts (the ThyroidOmics Consortium [20] and the UK Biobank [21]) (Table 1). GWAS summary data on FT4 and TSH were accessed from the ThyroidOmics Consortium, composed of 72,167 venous thromboembolism samples. Meanwhile, GWAS summary data on hyperthyroidism and hypothyroidism were obtained from the UK Biobank, comprising 337,159 venous thromboembolism samples.

### 2.2. Selection of Instrumental Variables

The screening criteria of FT4-, TSH-, hyperthyroidism-, and hypothyroidism-associated SNPs as instrumental variables were as follows: (i) *p* < 5 × 10^−8^; (ii) linkage disequilibrium was removed (kb = 10,000 and *r*^2^ = 0.01).

### 2.3. Two-Sample Mendelian Randomization Estimates

Five two-sample Mendelian randomization analysis methods containing inverse variance weighted (IVW), MR-Egger, weighted median, simple mode, and weighted mode were applied for estimating the casual effects of FT4, TSH, hyperthyroidism, and hypothyroidism on risk of venous thromboembolism. Among the five approaches, IVW was the main Mendelian randomization analysis to evaluate the causal effects because this approach is stable and accurate when directional pleiotropy is absent. All results were displayed as odds ratio (OR) and 95% confidence interval (CI), and *p* values < 0.05 were considered statistically significant. In addition, the results were visualized into forest and scatter plots of the relationships of FT4-, TSH-, hyperthyroidism-, and hypothyroidism-associated SNPs with risk of venous thromboembolism.

### 2.4. Heterogeneity and Pleiotropy Test

Mendelian randomization analysis exists heterogeneity because of the differences in platforms, experimental conditions, inclusion populations, and SNPs. Cochran's Q test of IVW analysis was conducted to detect heterogeneity. *p* values < 0.05 indicated significant heterogeneity.

### 2.5. Statistical Analysis

All statistical analysis was implemented utilizing Two-Sample MR package in R (version 3.6.2) [22].

## 3. Results

### 3.1. Selection of FT4-, TSH-, Hyperthyroidism-, and Hypothyroidism-Associated SNPs as IVs

**Figure 1:**
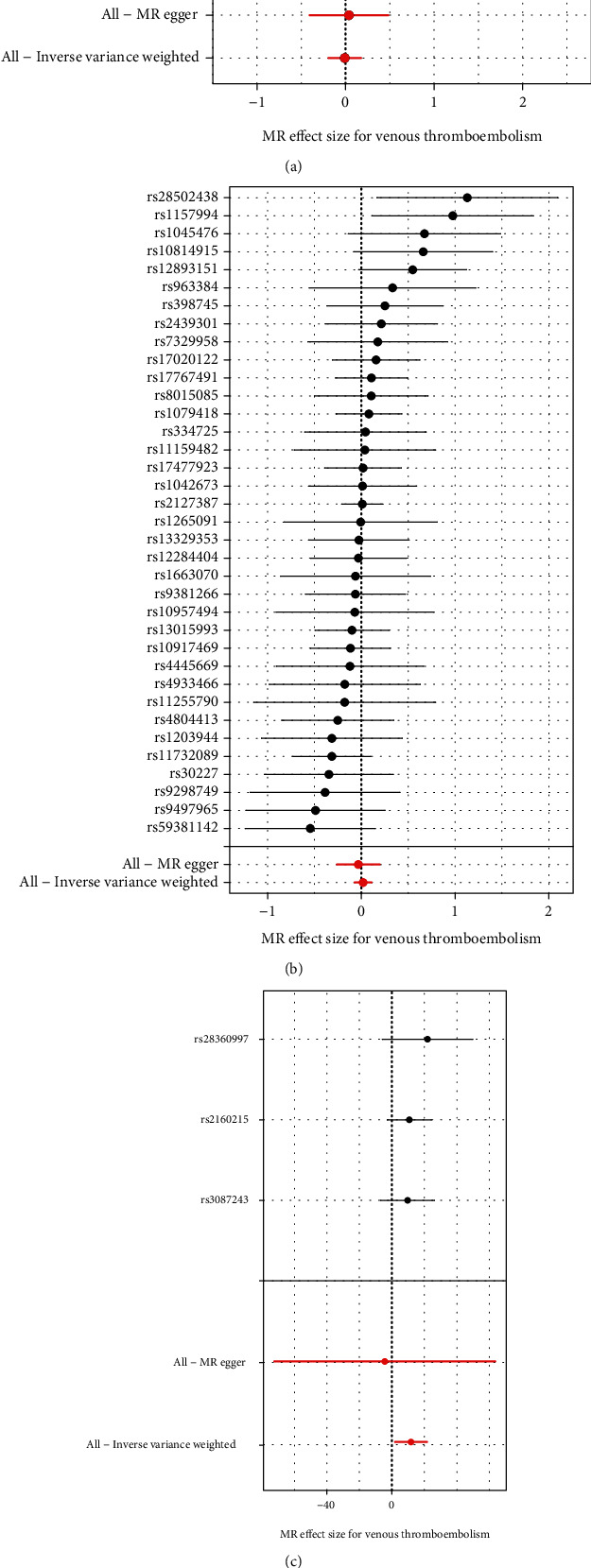
Forest plots for the association of FSH-, TSH-, hyperthyroidism-, and hypothyroidism-associated SNPs with risk of venous thromboembolism. (a) FSH, (b) TSH, (c) hyperthyroidism, and (d) hypothyroidism. In the forest plots, black points represent the log OR for venous thromboembolism per SD increase in FSH, produced using each FSH-associated SNP as an IV. Red points represent the combined causal estimates using all SNPs as an IV on the basis of MR-Egger and IVW methods. Horizontal line represents 95% CI of the estimates. Abbreviations: SNP: single nucleotide polymorphism; TSH: thyrotropin; OR: odds ratio; CI: confidence interval.

This study was conducted to investigate the causal relationships of FT4, TSH, hyperthyroidism, and hypothyroidism with venous thromboembolism via adopting two-sample Mendelian randomization. Based on the ThyroidOmics Consortium, we identified SNPs associated with thyroid function (FT4 and TSH). According to *p* < 5E − 8, LD *R*^2^ > 0.001, and distance < 10000 kb, we identified 15 FT4-associated SNPs as IVs, including rs10119187, rs10739496, rs10818937, rs10946313, rs11039355, rs113107469, rs17185536, rs2235544, rs225014, rs4149056, rs4842131, rs4954192, rs56069042, rs6785807, and rs9356988 (Table 2). Meanwhile, 36 TSH-associated SNPs were used as IVs, as follows: rs1042673, rs1045476, rs1079418, rs10814915, rs10917469, rs10957494, rs11159482, rs11255790, rs1157994, rs11732089, rs1203944, rs12284404, rs1265091, rs12893151, rs13015993, rs13329353, rs1663070, rs17020122, rs17477923, rs17767491, rs2127387, rs2439301, rs28502438, rs30227, rs334725, rs398745, rs4445669, rs4804413, rs4933466, rs59381142, rs7329958, rs8015085, rs9298749, rs9381266, rs9497965, and rs963384 (Table 3). Additionally, on the basis of UK Biobank, with the same criteria, we determined 3 hyperthyroidism-associated SNPs (rs2160215, rs28360997, and rs3087243; Table 4) as well as 79 hypothyroidism-associated SNPs as IVs (Table 5). As depicted in Figures 1(a)–1(d), forest plots displayed the estimates of each FT4-, TSH-, hyperthyroidism-, and hypothyroidism-associated SNP on venous thromboembolism.

### 3.2. Two-Sample Mendelian Randomization Analysis for Evaluating Causal Effects of FT4, TSH, Hyperthyroidism, and Hypothyroidism on Venous Thromboembolism

**Figure 2:**
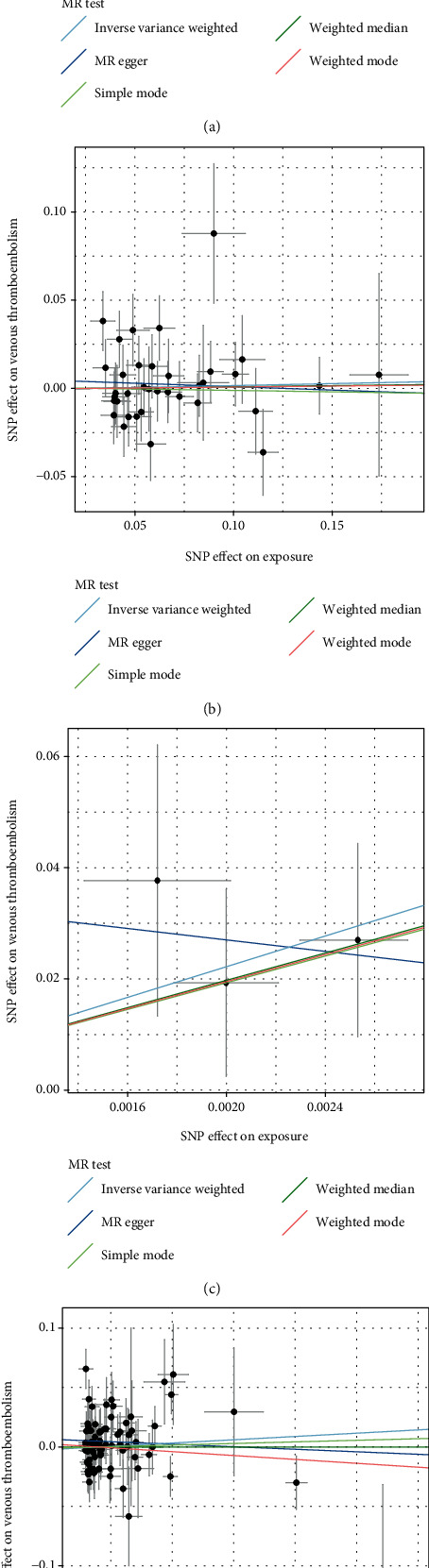
Scatter plots for the association of FSH-, TSH-, hyperthyroidism-, and hypothyroidism-associated SNPs with risk of venous thromboembolism. (a) FSH, (b) TSH, (c) hyperthyroidism, and (d) hypothyroidism. In the scatter plot, *x*-axis represents SNP effects on FSH, TSH, hyperthyroidism, and hypothyroidism (SD), and *y*-axis represents venous thromboembolism risk (log OR and 95% CI). The Mendelian randomization regression slopes of the lines denote the causal estimates utilizing five methods (IVW, MR-Egger, weighted median, simple mode, and weighted mode). Abbreviations: SNP: single nucleotide polymorphism; TSH: thyrotropin; OR: odds ratio; CI: confidence interval.

Five different two-sample Mendelian randomization analysis approaches (IVW, MR-Egger, weighted median, sample mode, and weighted mode) were conducted to assess the casual effects of FT4, TSH, hyperthyroidism, and hypothyroidism on risk of venous thromboembolism, as shown in Figures 2(a)–2(d). For the IVW analysis results, the OR of venous thromboembolism for hyperthyroidism was 1.124 (95% CI: 1.019-1.240; *p* = 0.019), without statistical significance for the MR-Egger, weighted median, sample mode, and weighted mode analysis results (Table 6). Meanwhile, no statistical significance from the five Mendelian randomization analysis approaches was found for FT4, TSH, and hypothyroidism on venous thromboembolism. The above data demonstrated that hyperthyroidism was a risk factor of venous thromboembolism.

### 3.3. Heterogeneity and Pleiotropy Test

**Figure 3:**
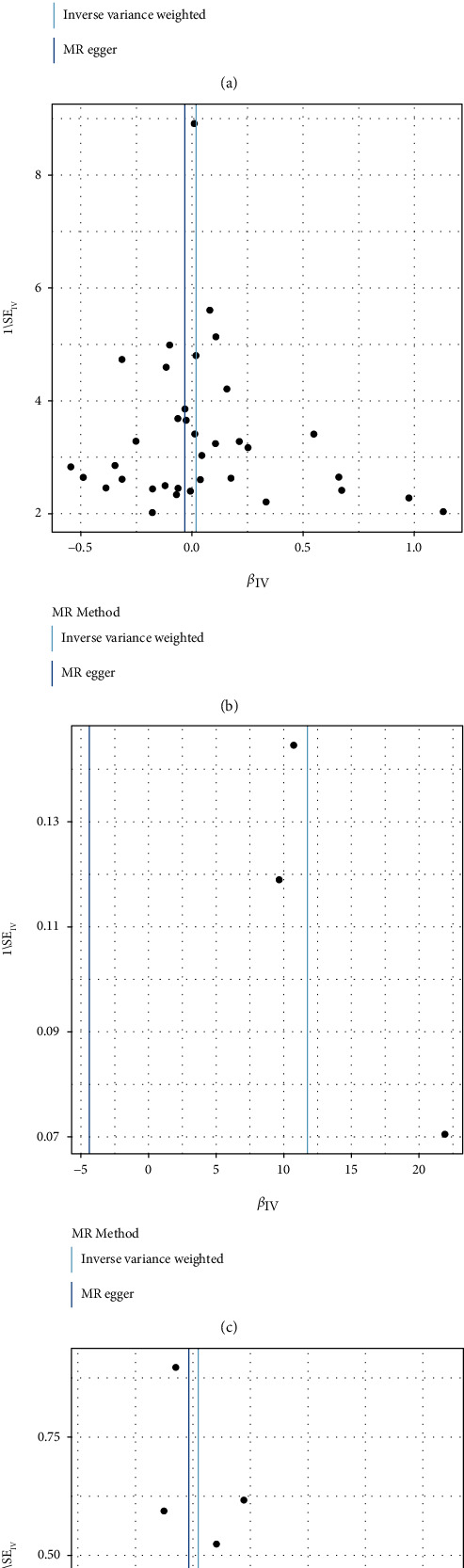
Funnel plots for the causal associations of FSH, TSH, hyperthyroidism, and hypothyroidism with risk of venous thromboembolism. (a) FSH, (b) TSH, (c) hyperthyroidism, and (d) hypothyroidism. Abbreviations: TSH: thyrotropin.

Through Cochran's Q test of IVW, we evaluated the heterogeneity and horizontal pleiotropy. A significant heterogeneity was found among FT4-associated SNPs on venous thromboembolism (*p* = 0.006). Moreover, there was no heterogeneity among TSH- (*p* = 0.574), hyperthyroidism- (*p* = 0.742), or hypothyroidism-associated SNPs (*p* = 0.209) on venous thromboembolism (Table 7). Further analysis showed that FT4- (*p* = 0.820), TSH- (*p* = 0.635), hyperthyroidism- (*p* = 0.721), and hypothyroidism- (*p* = 0.322) associated SNPs had no horizontal pleiotropy between FT4, TSH, hyperthyroidism, and hypothyroidism and risk of venous thromboembolism. As shown in funnel plots, when a single FT4-, TSH-, hyperthyroidism-, or hypothyroidism-associated SNP was used as an IV, the points representing the causality were relatively symmetrically distributed, suggesting that the cause was unlikely to be influenced by potential bias (Figures 3(a)–3(d)).

## 4. Discussion

Randomized controlled trials are the gold standard for causal inference in clinical research but are expensive and time-consuming and often difficult to implement due to ethical factors and subject constraints [23, 24]. Observational studies are relatively easy to implement and are widely applied for initial determination of etiology [25, 26]. Nonetheless, when there are reverse causal effects and potential confounding factors, the results of observational studies are often difficult to be recognized [27]. Mendelian randomization method provides an effective way to solve the above problems, using genetic variation as an instrumental variable effectively to avoid confounding factors caused by the environment [28]. Furthermore, compared with the immediate results obtained from randomized controlled trials, the exposure factors obtained from a genetic point of view are often accompanied for life and can even be passed on to the next generation [29]. Based on above evidence, we carried out the two-sample Mendelian randomization study to analyze the casual effects of FT4, TSH, hyperthyroidism, and hypothyroidism on venous thromboembolism.

The IVW method applies the ratio method to calculate causality estimates for individual instrumental variables and aggregates each estimate to perform a weighted linear regression to obtain an overall estimate [30]. However, this method cannot calculate the bias caused by pleiotropy, so an additional Cochran's Q test is needed to evaluate pleiotropy [31]. Our IVW analysis results showed that the ORR of venous thromboembolism for hyperthyroidism was 1.124 (95% CI: 1.019-1.240; *p* = 0.019), demonstrating the casual effect of hyperthyroidism not FT4, TSH, and hypothyroidism on venous thromboembolism. Additionally, there was no heterogeneity or horizontal pleiotropy in accordance with Cochran's Q test. Genome-wide association analysis uncovers that most variations in thyroid function are genetically determined [20]. In a nationwide population-based cohort study, hypothyroidism was in relation to higher risk of venous thromboembolism [32]. Decreased venous thromboembolism risk of hypothyroid patients treated with thyroxine replacement therapy was not investigated. However, our Mendelian randomization analysis showed that there was no causal effect of thromboembolism on venous thromboembolism. Another cohort study showed that TSH levels within the normal range were not associated with venous thromboembolism risk, but abnormal TSH levels were associated with moderate risk of venous thromboembolism [33]. Case-control studies have shown the significant association between serum TSH levels and increased risk of venous thromboembolism [34]. Consistently, TSH concentration was an independent risk factor of venous thromboembolism regardless of thyroid function [35]. Our causal analysis demonstrated that TSH was not casually associated with venous thromboembolism.

However, there are several limitations in our study. Firstly, the sample sets included in this study are all from the European region. Whether the research conclusions can be extended to other populations still needs further research to be proven. Secondly, there may be population sharing of sample sets from Europe, leading to overuse of genetic information.

## 5. Conclusion

Altogether, in the two-sample Mendelian randomization study with genome-wide association variants, our evidence demonstrated that hypothyroidism was casually associated with venous thromboembolism outcome. However, there was no evidence that TSH, FT4, and hypothyroidism had casual effects on venous thromboembolism. Despite this, further studies are required to validate the biological mechanisms underlying the casual association.

## Figures and Tables

**Table 1:** The detailed information of GWAS data included in Mendelian randomization analysis.

Exposure	Outcome	Detailed information of GWAS data	
		Consortium	Sample size
FT4	Venous thromboembolism	The ThyroidOmics Consortium	72,167
TSH	Venous thromboembolism	The ThyroidOmics Consortium	72,167
Hyperthyroidism	Venous thromboembolism	UK Biobank	337,159
Hypothyroidism	Venous thromboembolism	UK Biobank	337,159

Abbreviations: TSH: thyrotropin; FT4: free thyroxine.

**Table 2:** Univariate Mendelian randomization analysis for the effects of FT4 on venous thromboembolism risk.

SNP	EAE	OAE	EAO	OAO	Chr	Exposure			Outcome		
						Effect	SE	*p* value	Effect	SE	*p* value
rs10119187	T	C	T	C	9	0.0497	0.0085	5.00E-09	-0.0083	0.0225	0.713199
rs10739496	T	C	T	C	9	0.0777	0.0068	3.08E-30	-0.0262	0.0166	0.1152
rs10818937	C	T	C	T	9	0.0475	0.007	1.16E-11	-0.0129	0.0169	0.4454
rs10946313	T	C	T	C	6	0.0455	0.0068	2.21E-11	-0.0263	0.016	0.1003
rs11039355	C	T	C	T	11	0.0385	0.007	3.80E-08	0.0657	0.0172	0.0001329
rs113107469	T	C	T	C	18	0.1996	0.022	1.16E-19	0.007	0.0563	0.9009
rs17185536	T	C	T	C	6	0.0726	0.0081	3.16E-19	0.0111	0.0198	0.5749
rs2235544	A	C	A	C	1	0.1387	0.0065	4.99E-101	0.0193	0.016	0.2289
rs225014	T	C	T	C	14	0.0535	0.0067	1.40E-15	0.0084	0.0177	0.6346
rs4149056	C	T	C	T	12	0.0506	0.0089	1.31E-08	-0.0199	0.0198	0.315
rs4842131	C	T	C	T	9	0.1037	0.0075	1.76E-43	-0.0025	0.0159	0.8759
rs4954192	C	T	C	T	2	0.0409	0.0071	8.38E-09	0.0151	0.0161	0.3481
rs56069042	A	G	A	G	18	0.1061	0.0186	1.17E-08	-0.0334	0.0397	0.4005
rs6785807	G	A	G	A	3	0.059	0.0093	2.24E-10	-0.0021	0.018	0.9069
rs9356988	G	A	G	A	6	0.051	0.0073	2.82E-12	-0.0343	0.0166	0.0381698

Abbreviations: SNP: single nucleotide polymorphism; EAE: effect allele exposure; OAE: other allele exposure; EAO: effect allele outcome; OAO: other allele outcome; Chr: chromosome; SE: standard error.

**Table 3:** Univariate Mendelian randomization analysis for the effects of TSH on venous thromboembolism risk.

SNP	EAE	OAE	EAO	OAO	Chr	Exposure			Outcome		
						Effect	SE	*p* value	Effect	SE	*p* value
rs1042673	G	A	G	A	17	0.0546	0.0061	3.53E-19	7E-04	0.016	0.9638
rs1045476	A	G	A	G	16	0.049	0.0082	2.29E-09	0.033	0.0203	0.1045
rs1079418	A	G	A	G	6	0.1009	0.0066	9.21E-53	0.0081	0.018	0.6514
rs10814915	T	C	T	C	9	0.0421	0.0061	5.14E-12	0.0278	0.0159	0.0800995
rs10917469	A	G	A	G	1	0.1112	0.0085	4.15E-39	-0.0129	0.0242	0.5936
rs10957494	G	A	G	A	8	0.0402	0.0066	1.12E-09	-0.0028	0.0172	0.8688
rs11159482	T	C	T	C	14	0.0846	0.0129	5.45E-11	0.0032	0.0325	0.9226
rs11255790	C	T	C	T	10	0.041	0.0066	5.23E-10	-0.0073	0.0203	0.717999
rs1157994	G	A	G	A	17	0.09	0.016	1.86E-08	0.0878	0.0395	0.0262102
rs11732089	T	C	T	C	4	0.115	0.0076	1.00E-51	-0.0362	0.0243	0.1366
rs1203944	C	T	C	T	20	0.0509	0.0073	3.11E-12	-0.016	0.0195	0.4119
rs12284404	G	A	G	A	11	0.0667	0.0069	4.18E-22	-0.0021	0.0173	0.9014
rs1265091	T	C	T	C	6	0.0571	0.0086	3.15E-11	-4E-04	0.0238	0.987
rs12893151	C	A	C	A	14	0.0624	0.0078	1.24E-15	0.0342	0.0183	0.0614497
rs13015993	A	G	A	G	2	0.0818	0.0069	2.03E-32	-0.0082	0.0164	0.614899
rs13329353	T	C	T	C	15	0.0614	0.0065	3.52E-21	-0.0016	0.0168	0.9262
rs1663070	C	T	C	T	3	0.0463	0.007	3.73E-11	-0.0029	0.0189	0.8791
rs17020122	T	C	T	C	1	0.1044	0.0114	5.29E-20	0.0164	0.0248	0.5088
rs17477923	T	C	T	C	15	0.0826	0.0069	5.04E-33	0.0015	0.0172	0.9291
rs17767491	A	G	A	G	16	0.0883	0.0065	4.94E-42	0.0095	0.0172	0.5777
rs2127387	A	G	A	G	5	0.1435	0.0062	1.63E-118	0.0015	0.0161	0.9236
rs2439301	G	A	G	A	8	0.0587	0.0076	1.13E-14	0.0125	0.0179	0.4845
rs28502438	T	C	T	C	3	0.0338	0.0061	3.01E-08	0.0382	0.0166	0.0212902
rs30227	C	T	C	T	16	0.0468	0.0063	1.10E-13	-0.0162	0.0164	0.3225
rs334725	A	G	A	G	1	0.1737	0.0147	3.21E-32	0.0077	0.0573	0.8924
rs398745	C	A	C	A	14	0.052	0.0062	4.98E-17	0.0131	0.0164	0.4224
rs4445669	C	T	C	T	11	0.0397	0.0061	7.61E-11	-0.0048	0.0159	0.764599
rs4804413	T	C	T	C	19	0.0532	0.0062	9.43E-18	-0.0134	0.0162	0.4066
rs4933466	A	G	A	G	10	0.0395	0.0063	3.61E-10	-0.007	0.0162	0.666699
rs59381142	G	A	G	A	3	0.058	0.0076	2.32E-14	-0.0316	0.0205	0.1232
rs7329958	C	T	C	T	13	0.0439	0.0065	1.44E-11	0.0077	0.0167	0.644401
rs8015085	A	G	A	G	14	0.0671	0.0077	2.93E-18	0.0071	0.0207	0.7319
rs9298749	C	A	C	A	9	0.0393	0.0064	8.22E-10	-0.0152	0.016	0.3431
rs9381266	T	C	T	C	6	0.0726	0.007	3.34E-25	-0.0046	0.0197	0.8172
rs9497965	T	C	T	C	6	0.0444	0.0062	7.99E-13	-0.0217	0.0168	0.1971
rs963384	T	C	T	C	17	0.0351	0.0063	2.53E-08	0.0117	0.0159	0.4626

Abbreviations: SNP: single nucleotide polymorphism; EAE: effect allele exposure; OAE: other allele exposure; EAO: effect allele outcome; OAO: other allele outcome; Chr: chromosome; SE: standard error.

**Table 4:** Univariate Mendelian randomization analysis for the effects of hyperthyroidism on venous thromboembolism risk.

SNP	EAE	OAE	EAO	OAO	Chr	Exposure			Outcome		
						Effect	SE	*p* value	Effect	SE	*p* value
rs2160215	C	T	C	T	14	0.0025	0.0002	8.90E-31	0.027	0.0174	0.1207
rs28360997	A	G	A	G	6	-0.0017	0.0003	6.01E-09	-0.0377	0.0244	0.1224
rs3087243	A	G	A	G	2	-0.0020	0.0002	3.80E-21	-0.0193	0.0168	0.2505

Abbreviations: SNP: single nucleotide polymorphism; EAE: effect allele exposure; OAE: other allele exposure; EAO: effect allele outcome; OAO: other allele outcome; Chr: chromosome; SE: standard error.

**Table 5:** Univariate Mendelian randomization analysis for the effects of hypothyroidism on venous thromboembolism risk.

SNP	EAE	OAE	EAO	OAO	Chr	Exposure			Outcome		
						Effect	SE	*p* value	Effect	SE	*p* value
rs10036386	T	C	T	C	5	0.0032	0.0005	1.75E-09	-0.0022	0.016	0.8916
rs1032129	C	A	C	A	8	-0.0030	0.0005	2.67E-08	-0.0134	0.0161	0.4059
rs10424978	A	C	A	C	19	-0.0042	0.0005	7.21E-15	0	0.0162	0.998
rs1088898	T	G	T	G	17	0.0034	0.0006	4.22E-08	-0.0182	0.0173	0.2936
rs10956412	C	A	C	A	8	-0.0051	0.0007	5.84E-13	-0.0011	0.027	0.9661
rs11052877	G	A	G	A	12	-0.0058	0.0005	9.31E-27	-0.0127	0.0161	0.4304
rs111618453	A	G	A	G	13	0.0058	0.0006	4.33E-23	0.0019	0.0174	0.9144
rs11258303	A	C	A	C	10	0.0037	0.0006	5.59E-10	-0.0083	0.0167	0.6174
rs113229608	A	C	A	C	3	0.0065	0.0011	1.17E-09	-0.0584	0.0415	0.1588
rs11571297	C	T	C	T	2	-0.0086	0.0005	5.34E-61	-0.0176	0.0164	0.2833
rs11675342	T	C	T	C	2	0.0046	0.0005	1.67E-18	0.002	0.016	0.9001
rs11706511	G	A	G	A	3	0.0047	0.0008	2.47E-09	0.0356	0.0227	0.1166
rs11783023	T	C	T	C	8	-0.0033	0.0006	1.06E-08	0.0017	0.0164	0.9189
rs12117927	A	C	A	C	1	0.0033	0.0005	8.18E-10	-0.0295	0.0165	0.0749
rs12582330	T	G	T	G	12	-0.0042	0.0006	1.10E-12	-0.0128	0.017	0.452
rs12980063	G	A	G	A	19	-0.0034	0.0005	1.74E-10	-0.0138	0.0166	0.405
rs13090803	T	G	T	G	3	0.0051	0.0006	2.09E-15	0.0397	0.0229	0.0826
rs13145888	C	T	C	T	4	-0.0060	0.0006	2.45E-21	0.0351	0.0242	0.1465
rs13333582	C	T	C	T	16	0.0072	0.0013	1.80E-08	-0.0181	0.0413	0.6610
rs13399762	G	A	G	A	2	0.0067	0.0011	4.25E-09	0.0136	0.042	0.7470
rs142997491	G	A	G	A	16	0.0150	0.0024	2.81E-10	0.0296	0.0538	0.5826
rs1534430	T	C	T	C	2	-0.0040	0.0005	3.99E-14	-0.0029	0.0161	0.857
rs1549142	T	C	T	C	19	0.0041	0.0006	3.90E-11	0.0124	0.0186	0.5039
rs16903097	G	T	G	T	8	-0.0045	0.0008	3.97E-09	-0.0154	0.0248	0.5346
rs17020110	C	T	C	T	1	0.0041	0.0006	2.07E-12	-0.002	0.0189	0.9161
rs1790604	G	A	G	A	18	-0.0032	0.0005	5.49E-10	-0.0402	0.0159	0.01171
rs2111485	G	A	G	A	2	0.0038	0.0005	7.84E-13	0.0023	0.0161	0.8845
rs2123340	A	G	A	G	9	-0.0037	0.0005	7.76E-12	-0.0084	0.0167	0.6136
rs221786	C	T	C	T	7	0.0050	0.0008	1.53E-09	-0.0246	0.0222	0.2678
rs229540	G	T	G	T	22	0.0050	0.0005	8.83E-22	0.0251	0.0161	0.1204
rs2412974	T	C	T	C	22	-0.0031	0.0005	8.01E-09	0.0036	0.0162	0.8243
rs244672	T	C	T	C	5	-0.0046	0.0008	4.84E-09	-0.0153	0.0185	0.4085
rs2523483	G	T	G	T	6	-0.0066	0.0009	4.37E-13	-0.0254	0.0746	0.7332
rs2736191	G	C	G	C	6	-0.0094	0.0017	1.59E-08	-0.0548	0.0354	0.1212
rs2745803	G	A	G	A	20	-0.0037	0.0006	9.31E-09	0.021	0.0223	0.3448
rs28157	T	G	T	G	5	-0.0035	0.0006	4.16E-10	-0.0339	0.0171	0.04697
rs2823272	A	T	A	T	21	-0.0036	0.0006	2.03E-10	-0.0044	0.0183	0.8109
rs3184504	C	T	C	T	12	-0.0099	0.0005	1.10E-81	-0.044	0.0161	0.0062
rs3775291	T	C	T	C	4	-0.0040	0.0006	1.05E-12	0.0184	0.0171	0.2813
rs3850765	C	T	C	T	10	0.0031	0.0005	2.76E-09	0.0196	0.0174	0.2613
rs4081335	C	T	C	T	1	-0.0064	0.0012	3.55E-08	-4E-04	0.032	0.9889
rs4263621	A	G	A	G	6	0.0030	0.0005	1.48E-08	0.0036	0.0159	0.8189
rs4276275	T	C	T	C	4	0.0034	0.0005	5.84E-11	0.0039	0.0159	0.8079
rs4409785	C	T	C	T	11	0.0065	0.0007	4.31E-21	0.0102	0.0212	0.6326
rs4444866	T	C	T	C	4	-0.0032	0.0006	2.85E-08	0.0146	0.0175	0.4058
rs60600003	G	T	G	T	7	0.0050	0.0009	7.19E-09	-0.0184	0.0263	0.485
rs61759532	T	C	T	C	17	0.0042	0.0006	1.26E-11	-0.0068	0.0203	0.7381
rs62076510	G	T	G	T	17	0.0052	0.0007	3.46E-13	0.0343	0.021	0.1031
rs6426808	A	G	A	G	1	0.0033	0.0005	3.13E-10	-0.0108	0.0159	0.4976
rs654537	A	G	A	G	6	0.0060	0.0005	2.13E-29	-0.0029	0.0159	0.856
rs6584277	G	A	G	A	10	-0.0032	0.0005	8.79E-10	0.0229	0.0159	0.1488
rs66749983	T	A	T	A	13	0.0036	0.0006	1.39E-10	-0.0189	0.0167	0.2585
rs6679677	A	C	A	C	1	0.0201	0.0009	1.07E-122	-0.03	0.0224	0.1796
rs6833591	G	A	G	A	4	-0.0032	0.0005	6.10E-09	-0.0175	0.0193	0.3621
rs683763	T	G	T	G	10	0.0031	0.0006	2.57E-08	-0.0128	0.0162	0.4288
rs6992869	C	T	C	T	8	0.0030	0.0005	2.56E-08	0.0017	0.016	0.915
rs705702	G	A	G	A	12	0.0037	0.0005	1.96E-11	0.001	0.0172	0.9558
rs7090530	A	C	A	C	10	0.0042	0.0005	2.94E-15	-0.0055	0.0169	0.7451
rs71508903	T	C	T	C	10	0.0063	0.0007	3.21E-21	0.0202	0.0205	0.3242
rs736374	A	G	A	G	11	0.0041	0.0005	2.48E-14	0.005	0.0166	0.7631
rs7441808	G	A	G	A	4	0.0035	0.0006	3.53E-10	-1E-04	0.0176	0.9964
rs7582694	G	C	G	C	2	-0.0070	0.0006	2.61E-29	0.0086	0.0188	0.6460
rs761357	T	A	T	A	6	0.0033	0.0005	6.81E-10	0.0137	0.0167	0.4118
rs76428106	C	T	C	T	13	0.0271	0.0024	4.26E-30	-0.1052	0.0736	0.1528
rs7649344	C	T	C	T	3	-0.0030	0.0005	1.23E-08	0.003	0.0164	0.8565
rs76518703	G	A	G	A	6	-0.0101	0.0012	1.42E-16	-0.061	0.042	0.1464
rs7768019	G	C	G	C	6	-0.0081	0.0006	1.31E-41	0.0065	0.0178	0.7173
rs7905731	C	T	C	T	10	-0.0031	0.0005	2.66E-09	0.0207	0.0165	0.2095
rs8008961	T	C	T	C	14	-0.0034	0.0006	5.54E-09	0.0027	0.0173	0.8763
rs8043085	T	G	T	G	15	0.0043	0.0006	4.47E-12	-0.0021	0.0189	0.9122
rs8054578	G	A	G	A	16	-0.0036	0.0006	8.95E-09	0.0043	0.0209	0.8381
rs853303	G	A	G	A	8	-0.0037	0.0005	2.30E-12	-0.0191	0.0171	0.2658
rs911760	A	C	A	C	9	0.0042	0.0007	5.10E-10	0.0129	0.0195	0.5062
rs925489	T	C	T	C	9	0.0099	0.0006	1.52E-71	-0.0248	0.0166	0.1358
rs926103	C	T	C	T	1	-0.0030	0.0005	4.92E-08	-0.0657	0.0164	5.91E-05
rs9277569	T	C	T	C	6	0.0084	0.0008	1.36E-23	0	0.0226	0.9997
rs933243	A	C	A	C	6	-0.0056	0.0006	3.90E-24	-0.0104	0.0165	0.5268
rs9497965	T	C	T	C	6	0.0036	0.0005	5.19E-12	-0.0217	0.0168	0.1971
rs9815073	A	C	A	C	3	-0.0071	0.0006	1.94E-35	-0.004	0.0171	0.813

Abbreviations: SNP: single nucleotide polymorphism; EAE: effect allele exposure; OAE: other allele exposure; EAO: effect allele outcome; OAO: other allele outcome; Chr: chromosome; SE: standard error.

**Table 6:** Mendelian randomization analysis for estimating the associations between thyroid function and dysfunction and venous thromboembolism risk.

Exposure	Outcome	Ancestry	MR				
			Methods	nSNP	Beta	OR (95% CI)	*p* value
FT4	Venous thromboembolism	European	IVW	15	-0.009	0.991 (0.825, 1.190)	0.919
			MR-Egger	15	0.038	1.039 (0.667, 1.619)	0.869
			Weighted median	15	0.024	1.024 (0.858, 1.224)	0.789
			Sample mode	15	-0.024	0.976 (0.716, 1.330)	0.880
			Weighted mode	15	0.071	1.074 (0.886, 1.302)	0.481
TSH	Venous thromboembolism	European	IVW	36	0.019	1.019 (0.931, 1.115)	0.684
			MR-Egger	36	-0.032	0.968 (0.771, 1.216)	0.782
			Weighted median	36	0.011	1.011 (0.880, 1.161)	0.880
			Sample mode	36	-0.014	0.987 (0.792, 1.230)	0.950
			Weighted mode	36	0.009	1.009 (0.867, 1.175)	0.907
Hyperthyroidism	Venous thromboembolism	European	IVW	3	0.117	1.124 (1.019, 1.240)	0.019
			MR-Egger	3	-0.044	0.957 (0.488, 1.885)	0.920
			Weighted median	3	0.104	1.110 (0.998, 1.233)	0.043
			Sample mode	3	0.107	1.107 (0.971, 1.263)	0.268
			Weighted mode	3	0.103	1.103 (0.980, 1.254)	0.243
Hypothyroidism	Venous thromboembolism	European	IVW	79	0.470	1.601 (0.693, 3.699)	0.270
			MR-Egger	79	-0.361	0.697 (0.111, 4.386)	0.939
			Weighted median	79	0.001	0.999 (0.275, 3.634)	0.900
			Sample mode	79	0.228	1.256 (0.073, 2.588)	0.228
			Weighted mode	79	-0.558	0.572 (0.084, 3.899)	0.570

Abbreviations: FT4: free thyroxine; TSH: thyrotropin; IVW: inverse variance weighted; nSNP: number of single nucleotide polymorphisms; OR: odds ratio; CI: confidence interval.

**Table 7:** Heterogeneity and pleiotropy test results.

Exposure	Outcome	Heterogeneity			Pleiotropy
		Method	Cochran's Q	*p* value	*p* value
FT4	Venous thromboembolism	IVW	29.396	0.006	0.820
TSH	Venous thromboembolism	IVW	32.82	0.574	0.635
Hyperthyroidism	Venous thromboembolism	IVW	0.597	0.742	0.721
Hypothyroidism	Venous thromboembolism	IVW	87.849	0.209	0.322

Abbreviations: FT4: free thyroxine; TSH: thyrotropin.

## Data Availability

The datasets analyzed during the current study are available from the corresponding author on reasonable request.

## Abbreviations

SNP: Single nucleotide polymorphism
GWAS: Genome-wide association study
FT4: Free thyroxine
TSH: Thyrotropin
IVW: Inverse variance weighted
OR: Odds ratio
CI: Confidence interval.
